# Supplementary material for: Allostery Inhibition of BACE1 by Psychotic and Meroterpenoid Drugs in Alzheimer’s Disease Therapy
Source: Molecules. 2022 Jul 8;27(14):4372. doi: 10.3390/molecules27144372 (PMC9320338; doi:10.3390/molecules27144372)
Supplement: Supplementary file 1 [file molecules-27-04372-s001.zip › molecules-1527419-supplementary.pdf]

SUPPORTING INFORMATION

FOR

Allostery Inhibition of BACE1 by Psychotic and Meroterpenoid Drugs in Alzheimer's Disease Therapy

Samuel C. Ugbaja [1,*], Isiaka A. Lawal [2], Bahijjahtu H. Abubakar [3], Aganze G. Mushebenge [1], Monsurat M. Lawal [1] and Hezekiel M. Kumalo [1,*]

1 Discipline of Medical Biochemistry, School of Laboratory Medicine and Medical Science, University of
KwaZulu-Natal, Durban 4001, South Africa; aganzedar@gmail.com (A.G.M.); lawalmonsurat635@gmail.com (M.M.L.)

2 Chemistry Department, Faculty of Applied and Computer Science, Vanderbijlpark Campus, Vaal University of Technology, Boulevard, Vanderbijlpark 1900, South Africa; lawalishaq000123@yahoo.com

3 The Renewable Energy Programme, Federal Ministry of Environment, Aguiyi Ironsi Street, Way Maitama, Abuja 444, Nigeria; bahijjah@yahoo.com

* Correspondence: ugbajasamchii@yahoo.com (S.C.U.); kumaloh@ukzn.ac.za (H.M.K.)

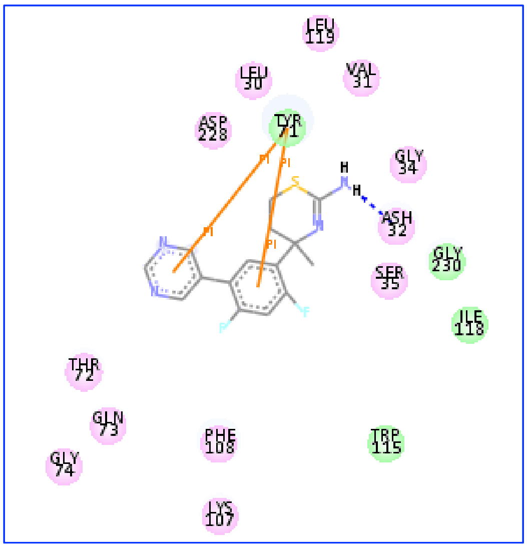

LY2811376

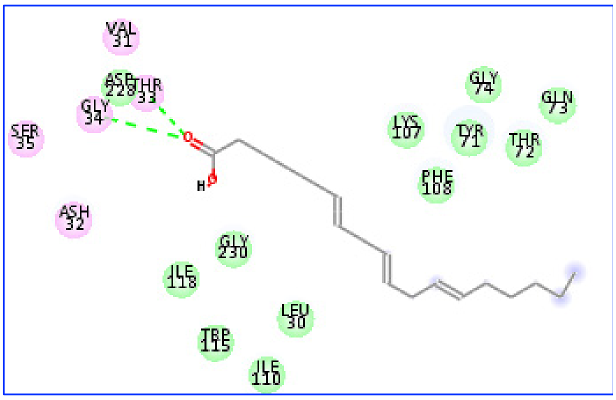

Gamma-linolenic acid

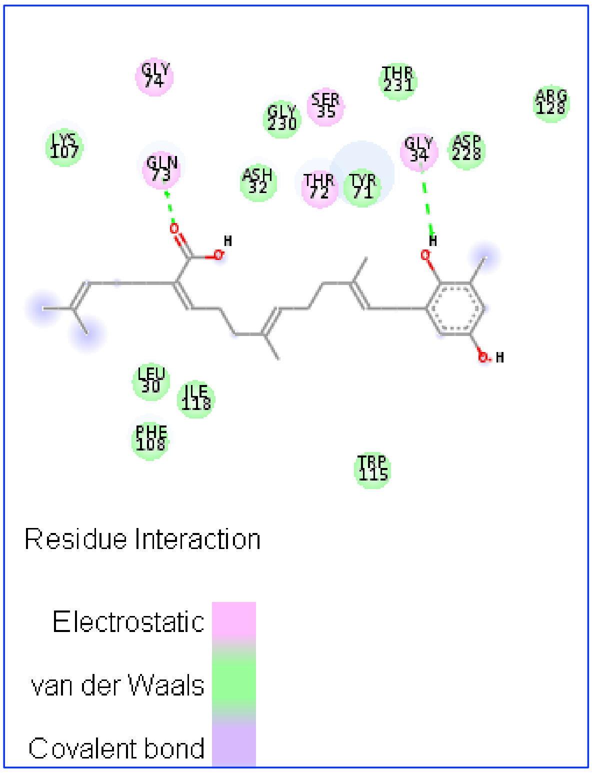

Sargahydroquinoic acid

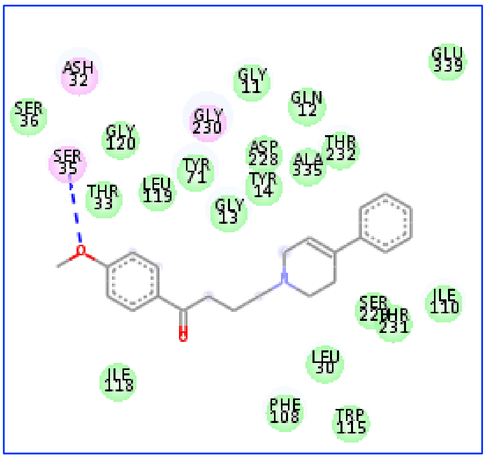

Anisoperidone

Figure S1: The interaction profile poses of the selected compounds in BACE1 active site. The dashed green and blue lines represent classical hydrogen bond and bridging water HB, respectively.

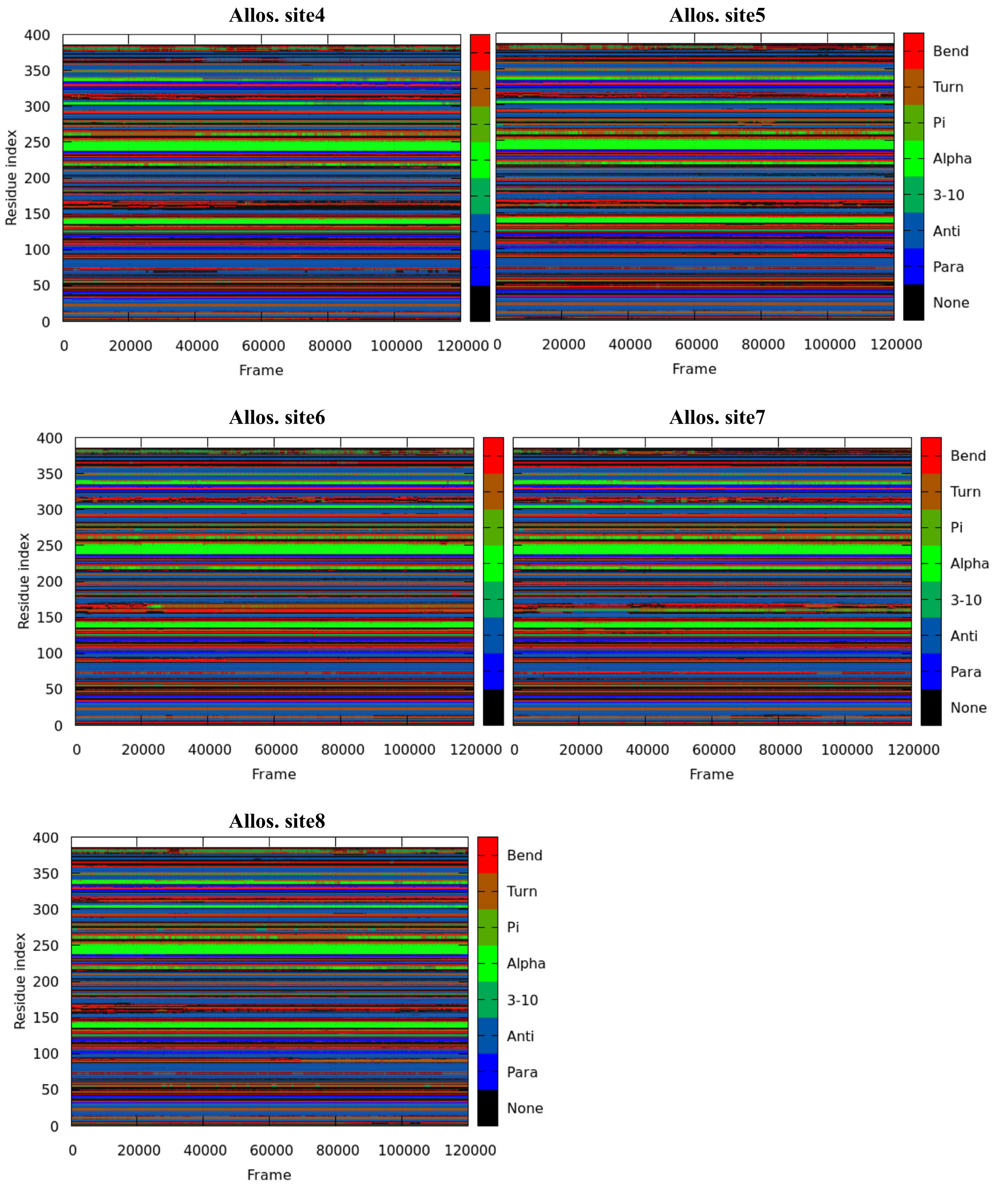

Figure S2: Protein secondary structure prediction over time 120 ns (120000 frames) for compound **1** binding to BACE1 allosteric sites.
